# T-Cell Aging-Associated Phenotypes in Autoimmune Disease

**DOI:** 10.3389/fragi.2022.867950

**Published:** 2022-04-25

**Authors:** Tuantuan V. Zhao, Yuki Sato, Jorg J. Goronzy, Cornelia M. Weyand

**Affiliations:** ^1^ Mayo Clinic Alix School of Medicine, College of Medicine and Science, Rochester, MN, United States; ^2^ School of Medicine, Stanford University, Stanford, CA, United States

**Keywords:** immune aging, autoimmune disease, rheumatoid arthritis, giant cell arteritis, tissue invasiveness, mitochondrial metabolism, treg aging, vasculitis

## Abstract

The aging process causes profound restructuring of the host immune system, typically associated with declining host protection against cancer and infection. In the case of T cells, aging leads to the accumulation of a diverse set of T-cell aging-associated phenotypes (TASP), some of which have been implicated in driving tissue inflammation in autoimmune diseases. T cell aging as a risk determinant for autoimmunity is exemplified in two classical autoimmune conditions: rheumatoid arthritis (RA), a disease predominantly affecting postmenopausal women, and giant cell arteritis (GCA), an inflammatory vasculopathy exclusively occurring during the 6th–9th decade of life. Pathogenic T cells in RA emerge as a consequence of premature immune aging. They have shortening and fragility of telomeric DNA ends and instability of mitochondrial DNA. As a result, they produce a distinct profile of metabolites, disproportionally expand their endoplasmic reticulum (ER) membranes and release excess amounts of pro-inflammatory effector cytokines. Characteristically, they are tissue invasive, activate the inflammasome and die a pyroptotic death. Patients with GCA expand pathogenic CD4^+^ T cells due to aberrant expression of the co-stimulatory receptor NOTCH1 and the failure of the PD-1/PD-L1 immune checkpoint. In addition, GCA patients lose anti-inflammatory Treg cells, promoting tissue-destructive granulomatous vasculitis. In summary, emerging data identify T cell aging as a risk factor for autoimmune disease and directly link TASPs to the breakdown of T cell tolerance and T-cell-induced tissue inflammation.

## Introduction

T lymphocytes are central players in the host defense against foreign pathogens, they recognize and remove malignant cells, coordinate tissue repair and control the appropriate recognition of autoantigens (Whiteside et al., 2018). As the host ages, T cell behavior and function undergo a series of changes that ultimately lead to the suppression of protective immunity and loss of the delicate balance between fighting foreign antigens and tolerating self-antigens (Kumar et al., 2018; Goronzy and Weyand, 2019). Aging in humans is typically associated with diminished protective immune responses to viral infections and impaired vaccine responses (Del Giudice et al., 2018; Gustafson et al., 2020; Poon et al., 2021). This is exemplified in the recent COVID-19 pandemic, where the mortality risk increased more than 10-fold in individuals older than 50 years of age when compared to young adults (Peng et al., 2020; Mogilenko et al., 2021a). Not only was advanced age a risk factor to succumb to SARS-CoV2 infection, but older individuals were also more likely to develop post-acute COVID-19 syndrome (Williamson et al., 2020; Sudre et al., 2021). Similarly, the effectiveness of most vaccines dramatically drops in older adults. Based on CDC statistics collected during the season of 2018–2019, vaccine effectiveness (VE) against Influenza A (H1N1) viruses reached 43% in 18–49-year-old adults; this number dropped to 30% in the 50–64 age group and to only 13% in people over the age of 65 years (https://www.cdc.gov/flu/vaccines-work/2018-2019.html).

The aging process is associated with a series of morbidities, most prominently type II diabetes, cardiovascular disease, and neurodegenerative disease. The progressive decline of the immune system is associated with a state of low intensity, smoldering inflammation often referred to as inflammaging that contributes to these morbidities (Franceschi et al., 2018a; Franceschi et al., 2018b; Furman et al., 2019). Rather unexpectedly, the risk of older individuals developing autoimmune disease is also increased, including autoimmune entities characterized by the excessive production of autoantibodies and the accumulation of autoreactive T cells (Mittelbrunn and Kroemer, 2021). A typical example is giant cell arteritis (GCA), an autoimmune and auto-inflammatory vasculopathy that affects the aorta and the large aortic branch vessels. Patients with GCA are typically older than 50 years and incidence rates peak during the 8th decade of life (Weyand and Goronzy, 2014). Likewise, the risk of being diagnosed with rheumatoid arthritis is highest in men and women who are older than 50 years of age and progressively increases during the last third of life (Weyand and Goronzy, 2021). The propensity of older individuals to develop tissue-destructive inflammatory disease was also noted in survivors of COVID-19 infection: the virus-imposed immune injury was associated with a multitude of chronic inflammatory conditions (Xie et al., 2022).

**FIGURE 1:**
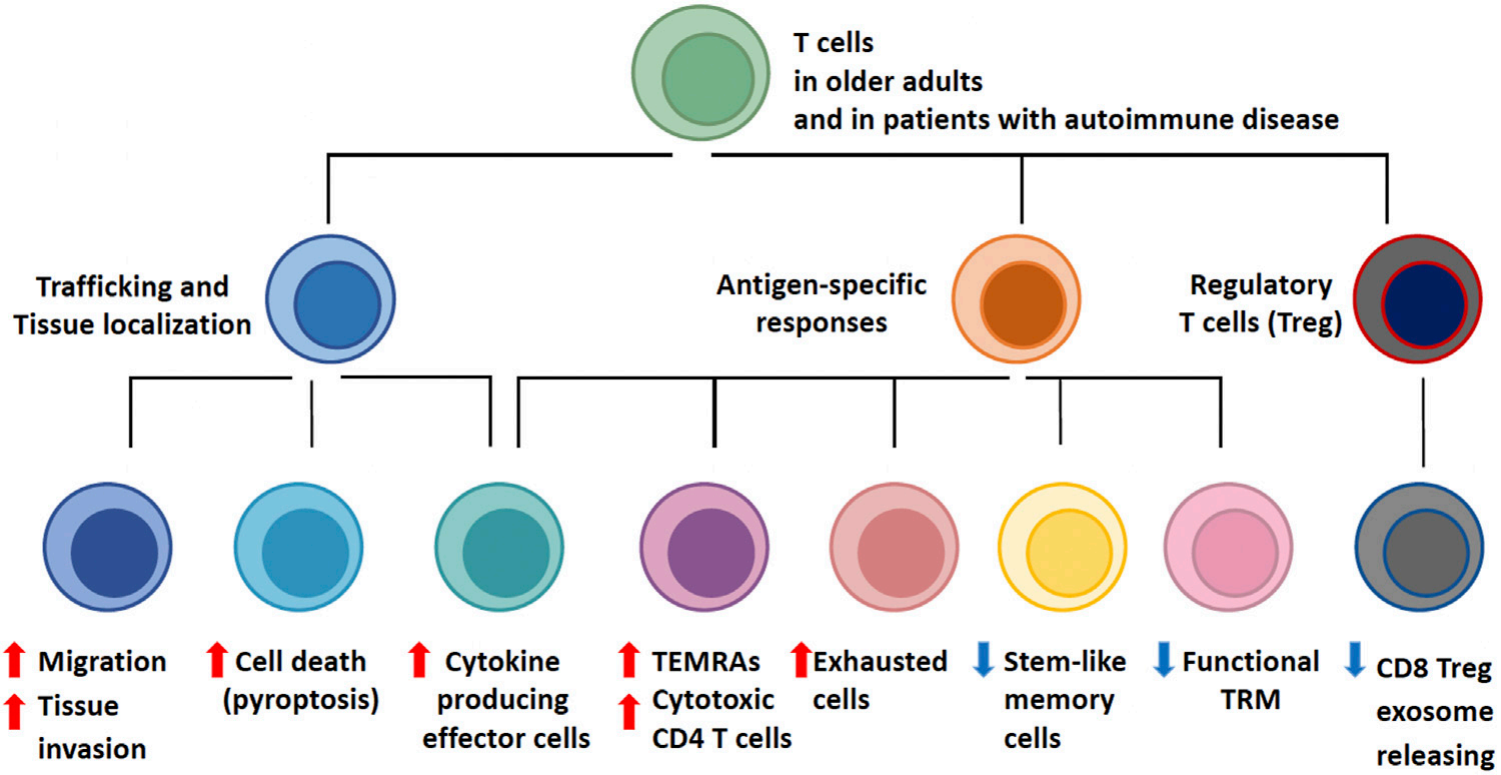
T-cell Aging-Associated Phenotypes (TASP). T cell aging leads to profound changes in the composition of the T cell pool. Major determinants of T cell aging include the dwindling generation of new T cells in face of continuous attrition and the stress imposed by acute and chronic antigen exposure, progressive differentiation and homeostatic proliferation. As naïve T cell populations shrink, the spectrum of phenotypically and functionally defined T cell subsets expands. Functional adaptations occur in three major domains: with age, T cells become more mobile and tissue invasive; with age, T cells differentiate into effector populations that are cytokine hyperproducers and possess cytotoxic functions; with age, regulatory T cells (Treg) deteriorate, allowing for unopposed effector responses. The assembly of T cell phenotypes in the aged T cell pool biases towards less specific and more inflammatory immunity. T cell aging-associated phenotypes (TASP) are enriched in patients with late-onset autoimmune diseases and have been implicated in pathogenic tissue inflammation, identifying the T cell aging process as a risk factor for the loss of self-tolerance.

The process of aging imposes a series of signatures on the T-cell compartment, which can be summarized as the T-cell aging-associated phenotype (TASP, Figure 1). A multitude of molecular mechanisms are involved in the generation of TASP, signifying the involvement of almost all fundamental cellular mechanisms that maintain homeostasis and allow the cell to adapt to acute and chronic stressors. T cells from older adults exhibit variations in cell cycle dynamics, energy production and utilization, handling of cellular garbage, cellular differentiation and lineage commitment, regulation of cell motility and trafficking, and engagement of cell death pathways (Sikora, 2015; Mogilenko et al., 2021a). As expected, multiple signaling pathways, transcription factor networks, metabolic programs, deviations in the DNA repair machinery and epigenetic regulations have been implicated in bringing about TASP. Remarkably, these phenotypes and the underlying defects have been evident in patients with autoimmune disease, supporting the concept that TASP predisposes the aging individual to lose self-tolerance and present with autoimmune tissue inflammation.

## T Cell Aging as a Predisposing Factor for Chronic Inflammatory Disease

### Functional (Mal)adaptations of Aging T Cells

The aging T-cell system adapts to major shaping forces: the production of new T cells dwindles due to bone marrow decline and thymic involution; post-thymic T cells have to deal with a world of antigens, including those produced by chronic infections; specialized lymphoid tissue hubs deteriorate; damaged DNA and proteins accumulate; memory and effector T cells reach exhaustion or enter the senescence program The T cell system responds dynamically, eventually acquiring a new organization and composition (Goronzy and Weyand, 2017). In older adults and in patients with selected autoimmune diseases, the T cell landscape is assembled of emerging subsets that have altered functional properties.

The aging process modulates the composition and functionality of the T cell compartment through multiple different avenues, but, essentially, a disproportionately high number of T cells from older adults and of T cells in RA patients have transitioned from being highly specific, clonally distributed, stem-like, and strictly controlled cells to more innate, end-differentiated, tissue invasive, short-lived effector cells that promote tissue inflammation. Such aged T cells are the result of integrated processes, creating a T-cell aging associated phenotype (TASP), that is, overall a hybrid state, combining loss-of-function and gain-of-function features.

**FIGURE 2:**
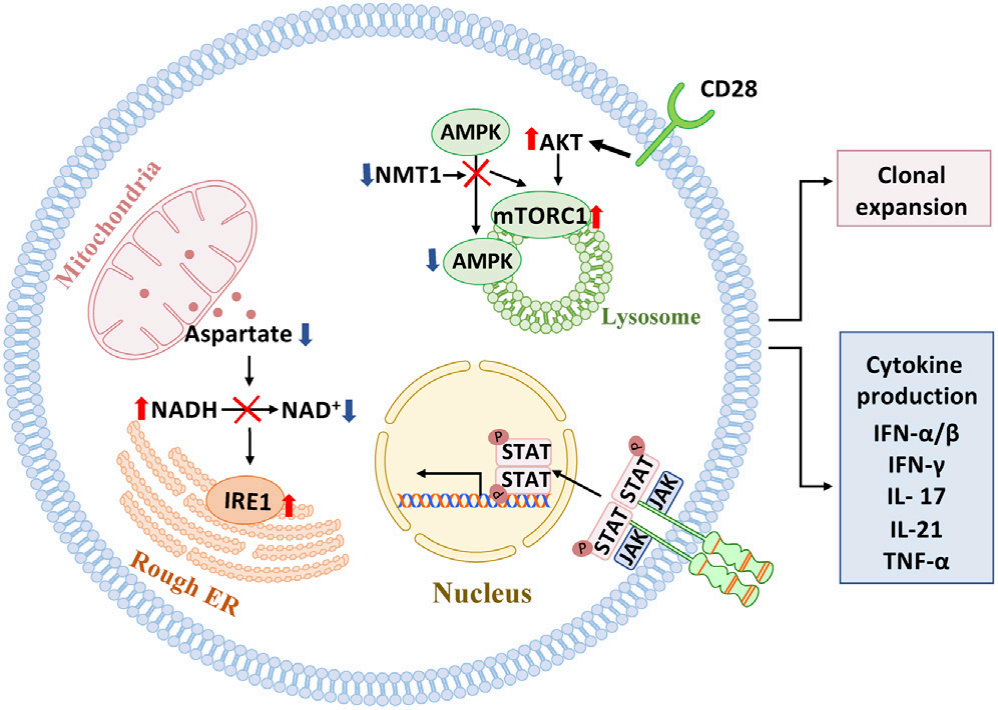
Signaling pathways in pathogenic T cells. Several signaling pathways have been implicated in mediating pathogenic T cell function in autoimmune tissue inflammation. In the autoimmune disease rheumatoid arthritis (RA), which affects individuals with a prematurely aged immune system, mitochondrial-ER communication is faulty due to insufficient generation of the amino acid aspartate, resulting in expansion of ER membranes and excessive production of the cytokine TNF-alpha. Also, in RA T cells, mitochondrial repair is impaired due to lacking activation of AMPK and unopposed activation of mTORC1 on the lysosomal surface. Mistrafficking of AMPK to the lysosome is a consequence of insufficiency in the enzyme N-myristoyltransferase 1(NMT1), which is responsible for posttranslational modification of AMPK. In giant cell arteritis, a strictly aging-associated autoimmune and autoinflammatory disease, persistent signaling through the JAK/STAT pathway and the CD28 pathway have been mechanistically connected to pathogenic immune responses in the inflamed tissues.

Loss-of-function is easier to conceptualize and connect to clinical outcomes: older adults lose clonal diversity, their memory T cells live shorter and have less “steminess,” and T-cell immunity lacks protective efficiency (Goronzy and Weyand, 2019). An important mechanism through which the aging immune system fails is T cell exhaustion (Akbar and Henson, 2011; Wherry and Kurachi, 2015; Blank et al., 2019; Zhao et al., 2020), a state of T cell malfunction that has been implicated in cancer and chronic viral infections. Gain-of-function involves persistent activation, abundant production of effector cytokines, tissue invasiveness and the acquisition of cytotoxic function by CD4 T cells (Elyahu et al., 2019). A functionally relevant abnormality in aged T cells is the emergence of strongly activated subpopulations, possibly a consequence of their inability to return to a state of resting. Unopposed activation may result from persistent activation of the mTOR pathway (Kim et al., 2018; Wen et al., 2019; Jin J. et al., 2021), hypersensitivity to activating stimuli surrounding the cells and global restructuring of DNA accessibility and histone synthesis and modification (Moskowitz et al., 2017; Kim et al., 2021). Gain-of-function also involves the transformation of CD4 T cells into cytotoxic effector cells (Nakajima et al., 2002; Niessner et al., 2006), a functional pathway mostly reserved for NK cells and CD8 T cells in young individuals. Accrual of killer CD4 T cells has been implicated as a disease mechanism in cardiovascular disease, where CD4 T cells can lyse both endothelial cells and vascular smooth muscle cells (Pryshchep et al., 2006; Sato et al., 2006), laying the ground for the immune-mediated damage of blood vessels, the major cause of age-related morbidity and mortality (Figure 2).

Here we will summarize what is currently known about TASP in autoimmune diseases and how aging-related phenotypes translate into T-cell-dependent tissue inflammation. Three phenotypes have been examined in detail: the process of tissue trafficking, T-cell-induced tissue injury and the death pathways of tissue-resident T cells.

### T-Cell Mobility and Invasiveness—How Aging T Cells Bring Disease to the Tissue

**FIGURE 3:**
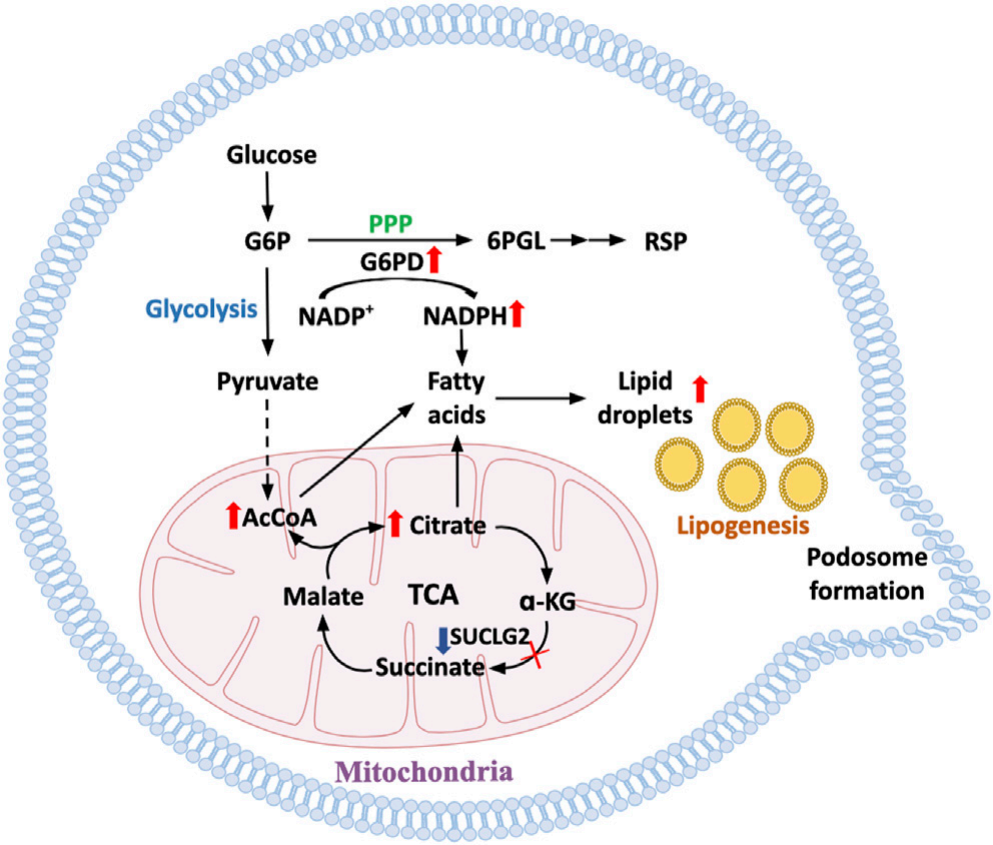
Metabolic pathways underlying enhanced cell motility in aged T cells. With progressive age, T cells become hypermobile and tissue invasive, facilitating their role in tissue inflammation. In T cells from prematurely aged patients with the autoimmune disease rheumatoid arthritis, mechanisms underlying the propensity of T cell invasiveness have been defined. Metabolic restructuring shifting glucose away from glycolytic breakdown to the pentose phosphate pathway promotes NADPH production and lipogenesis. Accumulation of intracellular lipids is enhanced by mitochondrial failure and disruption of beta oxidation. Lipid droplets serve as reservoirs for membrane formation, equipping the cells with invasive membrane structures and turning T cells into highly invasive, inflammatory effector cells.

Age-related autoimmune diseases, such as RA and GCA, occur because autoreactive T cells are recruited to and retained in the tissue niche (Weyand et al., 2018a; Weyand and Goronzy, 2021). In the case of RA, invading immune cells form tertiary lymphoid structures (Takemura et al., 2001a; Takemura et al., 2001b), an architectural feature that has been also associated with kidney aging (Sato et al., 2016; Sato et al., 2020; Sato et al., 2022). One hallmark of T cells isolated from RA patients is their ease of being transformed into a mobile T cell that rapidly slips into the synovial tissue to establish sophisticated extra-lymphoid structures, stimulating local stromal cells and functions as the key organizer of tissue inflammation. This pathogenic behavior has been linked to an aging phenotype (Schmidt et al., 1996; Weyand et al., 1998; Li et al., 2016) and to restructuring of cell-internal metabolic networks (Shen et al., 2017; Wu et al., 2020; Wu et al., 2021). Specifically, excess production of acetyl-CoA has been related to functional adaptations of the cellular cytoskeleton, changes in cellular shape, a bias towards uropod formation and altered placement of subcellular organelles (Wu et al., 2020). The underlying mechanism is the stiffening of the cytoskeletal network caused by hyperacetylation of tubulin. Altered membrane dynamics, associated with the swift formation of T cell podosomes seen in patient-derived T cells are a result of the restructuring of the cells’ lipid metabolism (Shen et al., 2017). Altogether, the pre-aged T cells that accumulate in patients with RA constitute a mobile, adaptable, moldable T cell force in which bioenergetics promotes tissue-invasive behavior (Figure 3).

The autoimmune and autoinflammatory disease GCA is strongly age-related, with no cases described in individuals younger than 50 years of age (Weyand and Goronzy, 2003). The metabolic profile of T cells infiltrating into the vessel wall is fundamentally different from that in RA patients (Weyand et al., 2018b; Gloor et al., 2022). Tissue entry of GCA CD4 T cells is facilitated by two mechanisms: Excessive production of MMP-9 in GCA macrophages that digest the basal membrane and prepare a path for invading T cells (Watanabe et al., 2018b) and a NOTCH-NOTCH ligand interaction between circulating CD4 T cells and microvascular endothelial cells (Wen et al., 2017). Aberrant expression of Notch 1 and Notch 4 receptors on circulating CD4 and CD8 T-cells, respectively, is one of the hallmark abnormalities in GCA (Piggott et al., 2011; Jin K. et al., 2021). Notably, NOTCH is recognized as one of the major metabolic regulators, considered to be critically involved in setting activity of the glycolytic pathway (De Bock et al., 2013; Kuwabara et al., 2018); the ability of pathogenic CD4 T cells to make their way into a peripheral tissue site appears ultimately controlled by the metabolic status of disease-inducing T-cells. Once in the tissue, the T cells form granulomatous arrangements with macrophages, with no significant B-cell component (Weyand and Goronzy, 2013). How Notch signaling accesses the migratory machinery of T-cells, including the cytoskeleton and membrane formation, remains to be investigated.

How the tissue environment itself becomes a “partner-in-crime,” enabling and fostering the entrance and retention of proinflammatory T cells is much less understood. Tissue aging may well emerge as a shaping factor in facilitating the attraction of inflammatory effector cells (Hernandez-Segura et al., 2018; Calcinotto et al., 2019) and the metabolic microenvironment will be a major determinant in the fate decisions of such cells.

### Pro-Inflammatory T-Cell Death—The Power of the Dead

**FIGURE 4:**
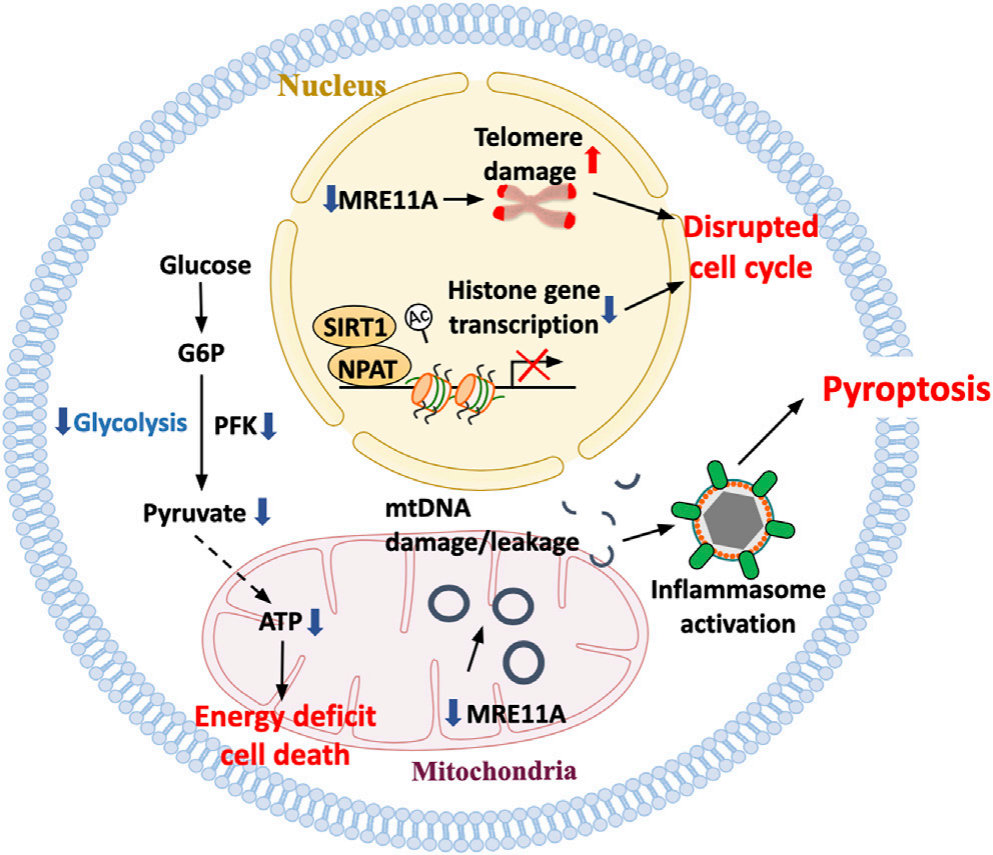
Death pathways in aged T cells. With age, T cells are at increasing risk to die, aggravating the need for T cell replenishment but also exposing the tissue environment to an inflammatory nidus. Misregulation of histone production as well as accumulation of damaged telomeric ends have been implicated in disturbing proper cell cycle progression and inducing T-cell death. Mitochondrial insufficiency serves as a major cause of lacking ATP production, depriving the cells of the bio-energy needed for survival. The inability of aging T cells to maintain stability of the mitochondrial genome has been mechanistically linked to the leakage of mtDNA into the cytosol, the activation of the inflammasome and the triggering of pyroptotic death. T cell pyroptosis purges T-cell reservoirs in lymph nodes and induces aggressive inflammation in peripheral tissues sites.

T cells are long-lived, as exemplified by the persistence of vaccine responses from childhood into high age. Humans possess about half a trillion T cells that are born in the bone marrow, selected in the thymus, and are surviving in lymphoid organs. T cell demise has to be matched by T cell regeneration to maintain the pool intact. During the first two decades of life, the thymus is the major site of T cell production, but thymic involution progressively limits the generation of new T cells after the age of 20 years. During the second half of life, humans need to replenish their T cells by replicating post-thymic T cells. Given that T cells are somatic cells, their replicative ability is limited and eventually clonotypes will disappear from the repertoire (Qi et al., 2014; Goronzy et al., 2015). When encountering antigen, T cells have enormous expansion capacity, associated with a complex differentiation program and also replicative stress. Accumulation of damaged DNA and progressive telomeric shortening will eventually limit the T-cell lifespan, with the type of death of considerable impact on the surrounding tissue (Figure 4).

Human CD4 T cells accumulate damaged DNA over lifetime, with acceleration of this process in RA patients (Li et al., 2018). Apoptotic T-cell loss in the patients may aggravate replicative stress, further accelerating the immune aging process. The major underlying defect has been identified as the transcriptional repression of the DNA repair kinase ataxia telangiectasia mutated (Weyand et al., 2018b). Additional attrition of RA T cells has been attributed to aberrant expression of the DNA repair enzyme DNA-protein kinase catalytic subunit (DNA-PKcs), an enzyme required for non-homologous-end-joining (Shao et al., 2010). T-cells isolated from RA patients high in DNA-PKcs are lost through a p53-independent death pathway. Additional defects in the DNA repair machinery of RA T cells involving the DNA repair nuclease MRE11A have been identified as a key factor determining their life span and also their functional behavior (Li et al., 2016; Li et al., 2019). Specifically, deficiency of MRE11A at the telomeric DNA ends has been associated with telomeric fragility and early aging of the T cells. Forced loss-of-function of MRE11A leads to the induction of the CD57^+^ phenotype, a well-recognized aging marker in T cells (Li et al., 2016). Overall, deficiencies in the DNA repair machinery are a hallmark of the prematurely aged T cells of RA patients. In functional studies, genetic or pharmacological inhibition of MRE11A caused aggressive inflammation in synovial tissue, resembling the disease process seen in patients with rheumatoid arthritis and lending strong support to the concept that unrepaired DNA and fragile telomeres are pinnacle abnormalities in not only driving the aging process but also the functional preferences of autoreactive T cells.

Difficulties in maintaining genome stability in aging T cells are not limited to the nucleus but also affect maintenance of the DNA in the mitochondria (Li et al., 2019). T cells derived from RA patients lack sufficient MRE11 in mitochondria, which has been mechanistically linked to deficiencies in the mitochondrial electron transport chain, oxidative damage of mtDNA and diminished ATP production (Li et al., 2019). Functional consequences include the leakage of mtDNA fragments into the cytosol where they are recognized as danger-associated molecular patterns, triggering assembly of the inflammasome and activation of caspase 1. The outcome is pyroptotic T-cell death, a death form that per se is strongly inflammatory and is coupled with the release of IL-1 and IL-18. Pyroptotic T-cells, a pathology previously described to occur in HIV-infected individuals who developed severe T-cell lymphopenia, are present in high abundance in the lymph nodes of RA patients. Here, RA and HIV infection appear to share the acceleration of the T-cell aging process. Pyroptotic RA T cells functioned as potent inflammation initiators in human synovial tissue.

A TASP described in older adults is the activation-induced increased expression of CD39, a nucleoside triphosphate diphosphohydrolase (NTPDase) that catalyzes the hydrolysis of ATP and ADP to the monophosphate form (AMP) (Antonioli et al., 2013; Timperi and Barnaba, 2021). In the context of CD73 expressing cells, AMP will be further metabolized to adenosine. These aged T-cells are therefore capable of depleting ATP and creating an adenosine-rich microenvironment, generally considered to be highly immunosuppressive. At the same time, CD39^+^ T-cells are highly effective as cytokine-producing effector T-cells and inflammatory effector cell differentiation is even enhanced by adenosine (Cao et al., 2020). These cells are prone to die (Fang et al., 2016; Cao et al., 2020), the consequences of their death on their immediate environment are currently unclear.

In essence, the inability to maintain a stable genome in the nucleus and the mitochondria renders aged T cells susceptible to death and the choice of death pathway has profound implications for the imprint that such dying T cells leave in the tissue.

### Cytokine-Hyperproducing T Cells—The Fallout of Mitochondrial DNA Instability

T cells of RA patients have a signature of premature aging, manifesting with age-inappropriate telomere shortening (Koetz et al., 2000; Schonland et al., 2003). The phenotype of premature telomeric loss is shared by myeloid cells and lymphocytes (Colmegna et al., 2008), suggestive for bone marrow stress as an upstream mechanism. The prematurity of aging in myeloid and lymphoid cells makes RA an excellent model system to define and characterize relevant molecular processes that mediate immune aging (Li et al., 2018), to begin to understand how the immune system adapts to aging imposed pressures and how aged immune cells contribute to tissue inflammation.

A critical defect leading to premature immune aging in RA has been assigned to the DNA repair machinery. T cells in RA patients have low amounts of the DNA repair kinase ataxia telangiectasia mutated (ATM), accumulate damaged DNA and are at increased risk to die prematurely (Shao et al., 2009; Shao et al., 2010). Insufficiency of DNA repair at telomeric ends has been implicated in telomere fragility (Li et al., 2016). The inability to properly maintain DNA stability extends to the mitochondrial genome, where the loss of the ATM partner molecule MRE11A leads to accumulation of damaged mtDNA. The leakage of such damaged DNA into the cytoplasm results in the triggering of the inflammasome (Li et al., 2019). In essence, instability of both nuclear and mitochondrial DNA lies at the heart of the prematurity of T-cell aging in RA. Insufficiency of mtDNA repair has been linked to two major outcomes that are ultimately responsible for autoimmune tissue inflammation. Cytoplasmic leakage of mtDNA activates caspase-1, facilitates inflammasome assembly and pyroptotic T-cells death, both in the lymph node and the synovial space. Excessive T-cell loss likely imposes replicative stress, aggravating telomeric shortening and mitochondrial dysfunction.

Erosion of mitochondrial fitness directly promotes the expansion of CD4^+^ T cells that transform into cytokine hyperproducers (Qiu et al., 2021; Weyand and Goronzy, 2021) (Figure 2). Mitochondrial insufficiency has profound implications for how the prematurely aged RA T cells respond to antigenic stimuli and release copious amounts of proinflammatory cytokines, including TNF, interferon-γ, IL-17 and IL-21 (Weyand and Goronzy, 2017; 2020; 2021). Production and secretion of the key cytokine TNF results from the inappropriate expansion of endoplasmic reticulum membranes and the enhancement of co-translational translocation caused by the lack of mitochondrial aspartate production (Wu et al., 2020). Failure of the mitochondrial electron transport chain and reversal of the TCA cycle result in accumulation of acetyl-CoA (Wu et al., 2020), hyperacetylation of cytoskeletal proteins, deposition of neutral lipids as lipid droplets and acceleration of membrane formation, enabling the T cells to form invasive membrane structures (Shen et al., 2017).

**FIGURE 5:**
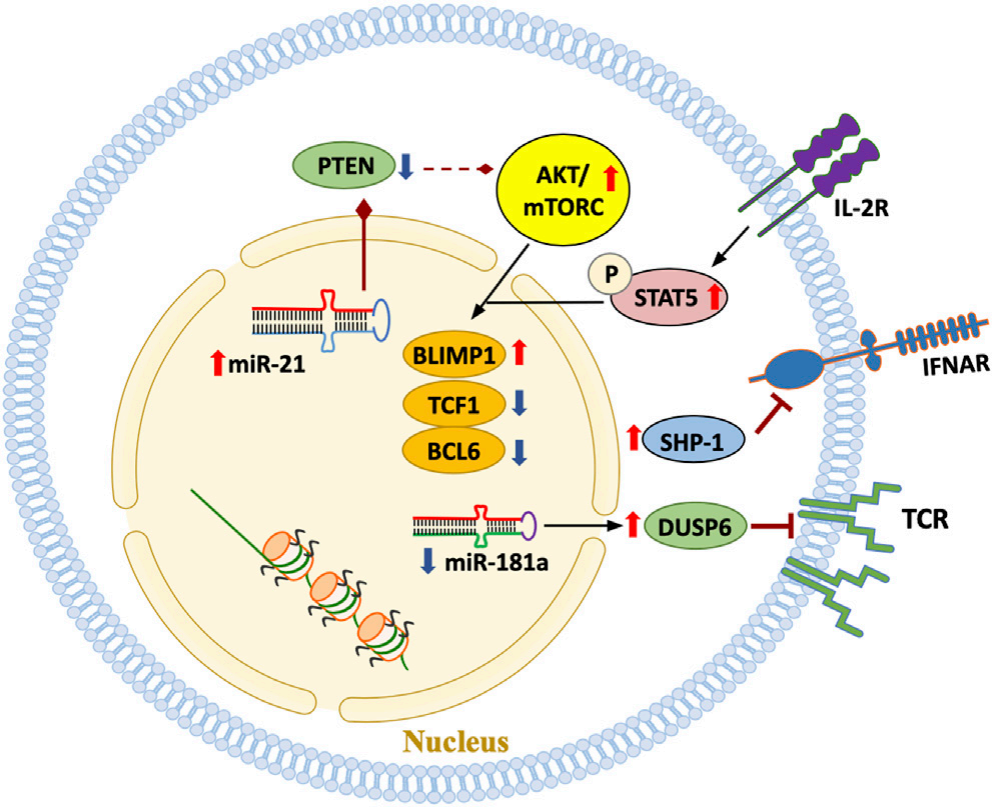
Signaling pathways in aged T cells. A hallmark of the T cell pool in the older adult is the emergence of T cell phenotypes that are functionally highly differentiated, possibly due to the replicative stress imposed by chronic antigenic stimulation and the cytokine milieu. Some T cell subpopulations progress towards end-differentiation. Multiple signaling pathways outlined in this scheme have been implicated in mediating cytokine- and antigen-derived signals and in determining the response pattern of the T cell.

Declining mitochondrial competence equally affects extramitochondrial metabolism. Glycolytic activity in RA is suppressed due to transcriptional repression of the key glycolytic enzyme phosphofructokinase (Yang et al., 2013). Functional consequences include a reduction in pyruvate as well as lactate and shifting of glucose towards the pentose phosphate pathway (Yang et al., 2016). This favors NADPH production and, together with impaired mitochondrial activity, supports reductive stress (Weyand et al., 2018b). Overall, deficiencies in maintaining the stability of mtDNA lead to a T cell, that is, metabolically reprogrammed, favors anabolic over catabolic processes, has biosynthetic precursor molecules at its disposal and turns into a short-lived cytokine hyperproducer that rapidly invades tissues sites where it induces and sustains tissue inflammation. The reprogramming of mitochondrial and extramitochondrial metabolism and the divergence towards the rapid generation of cellular offsprings relies on the inappropriate activation of the mTOR pathway. Several signaling pathways are recalibrated with age (Figure 5); in particular, increased mTORC1 activity is a hallmark of the aged T cell. mTORC1 activity has been directly linked to the cytokine production of RA T cells and their functionality as pathogenic effector cells in the synovial tissue (Weyand et al., 2017; Weyand and Goronzy, 2020). Mechanistically, inappropriate mTORC1 activity results from the failure of AMPK-imposed mTOR control; a defect attributed to faulty posttranslational modification of AMPK in RA T cells, which fail to recruit AMPK to the lysosomal surface (Wen et al., 2019).

Available data suggest that the linkage between genome instability, mitochondrial failure, metabolic reprogramming and pathogenic effector functions is disease-specific for RA. In GCA, a quintessential aging-associated autoimmune disease, T cells and macrophages display a distinct metabolic signature (Watanabe et al., 2017; Watanabe et al., 2018a; Watanabe et al., 2020). Precisely, pathogenic T-cell effector functions in GCA patients depend on a combination of JAK/STAT signaling (Zhang et al., 2018) and unopposed mTOR hyperactivity (Wen et al., 2017). CD28-dependent signaling in tissue-infiltrating T cells secures replenishment of vasculitogenic T cells by biasing the metabolic machinery towards glycolytic flux (Zhang et al., 2019). Disease specificity of molecular processes that link T-cell aging to autoimmune tissue inflammation provides strong support for the concept that a multitude of abnormalities in adaptive immunity can occur as the host ages predisposing the host to develop late onset autoimmunity.

### Cytotoxic CD4^+^ T Cells—Expanding the Possibilities

Although direct cytotoxic effector functions of T cells are thought to reside within the CD8^+^ T cell compartment, acquiring the competence to lyse target cells is a hallmark of T cell aging, bringing CD4 T cells into the realm of killer cells. This is exemplified in supercentenarians (older adults >110 years), who typically have high frequencies of clonally expanded GZMK^+^ CD4^+^ cytotoxic T cells among circulating lymphocytes (Hashimoto et al., 2019). Such GZMK^+^ CD4^+^ cytotoxic T cells exhibit almost identical transcriptome signatures to those of CD8^+^ cytotoxic lymphocytes and produce abundant amounts of IFN-γ, granzyme B, and perforin. These effector molecules are all regulated by EOMES, T-BET, and RUNX3 (Elyahu et al., 2019; Hashimoto et al., 2019). Notably, the functional activity of these transcription factors and the dependent effector molecules do not decline with age. Like other TASP, these cytotoxic CD4^+^ T cells may have dual functional impact: 1) tissue damaging effects, contributing to inflammaging and autoimmune tissue inflammation; 2) host protection. Cytotoxic CD4^+^ T cells are detected more abundantly in older patients with mild COVID19 compared with those with severe COVID19 (Arthur et al., 2021), raising the possibility that these cells assist in antiviral immunity (Juno et al., 2017).

Evidence has been provided that cytotoxic CD4^+^ T cells have a role in disease. Specifically, a potentially detrimental role has been proposed in the autoimmune entities IgG4 related disease (IgG4RD) and systemic sclerosis where they have been implicated in the production of pro-fibrotic cytokines and the induction of endothelial cell apoptosis, respectively (Mattoo et al., 2016; Maehara et al., 2020). Also, cytotoxic CD4^+^ T cells have been associated with idiopathic pulmonary fibrosis, an age-related lung disease. Support for a pathogenic role of such killer CD4 T cells comes from studies in coronary artery disease, a prototypic age-associated morbidity. Molecular analysis of atherosclerotic plaque infiltrating CD28^null^CD4^+^ T cells has demonstrated their clonal expansion, their accumulation in unstable plaques, their bias towards producing large amounts of IFN-γ, and their cytotoxic ability (Liuzzo et al., 1999; Liuzzo et al., 2000; Liuzzo et al., 2001; Nakajima et al., 2002; Nakajima et al., 2003; Pryshchep et al., 2006; Sato et al., 2006). Evidence implicating clonally expanded cytotoxic CD4^+^ T cells in the killing of endothelial cells and vascular smooth muscle cells has linked these aged effector T-cells directly to the process of plaque destabilization. A recent study employing scRNAseq analysis to examine immune cell populations within atherosclerotic plaques has confirmed the presence of CD4^+^ cytotoxic T cells in the tissue lesion (Depuydt et al., 2020). With atherosclerosis being one of the lead manifestations of aging, CD4^+^ T cells that have acquired cytotoxic capabilities may represent a critical bridge between immune aging and age-related disease.

 Cytotoxic effector CD8^+^ T cells may be even more substantially affected by aging than CD4^+^ T cells, given that the CD8 compartment is overall more susceptible to aging-imposed changes (Goronzy and Weyand, 2019). Loss of circulating naïve CD8^+^ T cells with aging is more severe than those of CD4^+^ T cells, and reduced numbers of naïve circulating CD8^+^ T cells are the most consistent and reliable marker of immune aging in healthy older adults independent of comorbidities (Goronzy and Weyand, 2019). A recent comprehensive study utilizing scRNAseq and scTCRseq of murine tissues has revealed fewer naïve CD8^+^ T cells across four tissues and has identified a distinct age-associated exhausted GZMK^+^CD8^+^ T cell population (Mogilenko et al., 2021b). These cells are characterized by the expression of PD1, LAG, and TIGIT, all of which are positively regulated by the transcriptional factor TOX (Scott et al., 2019). Age-associated GZMK^+^CD8^+^ T cells gradually accumulate in multiple tissues. Remarkable, despite the high expression of inhibitory molecules, these aged GZMK^+^CD8^+^ T cells rapidly respond to TCR stimulation and produce copious amounts of pro-inflammatory molecules, including GZMK and CCL5. In this study, only GZMK- and not GZMB- expressing CD8^+^ effector memory T cells proportionally increased in abundance with age relative to total CD8 T cells and correlated with markers of inflammaging, such as IL-6 and TNF (Mogilenko et al., 2021b). An important difference of age-associated GZMK^+^CD8^+^ T cells between humans and mice is that those in humans cannot be identified based on PD1 expression (Mogilenko et al., 2021a; Arthur et al., 2021; Mogilenko et al., 2021b). This discrepancy between mice and humans remains unexplained. It has been speculated that the exposure to multiple pathogens as it typically occurs in humans might be the underlying mechanism (Mogilenko et al., 2021b).

### Exhausted T-Cells—A Link to Iatrogenic Autoimmunity

If the term “exhausted T cells” is strictly used to describe effector T cells with increased expression of several inhibitory receptors and reduced capacity to secrete cytokines, the evidence for age-dependent T cell exhaustion is relatively limited. However, exhaustion may not involve the complete absence of function, and such defined exhausted T cells are often observed in patients with autoimmunity and may have pathogenic relevance.

Exhaustion may be the appropriate term to describe CD8^+^ T cells that accumulate in patients with juvenile idiopathic arthritis (JIA), a childhood autoimmune condition. JIA patients have been reported to have shortening of telomeres already in the naïve T cell compartment and reduced proliferative capacity in the entire T cell pool (Dvergsten et al., 2013). Although these patients are chronologically young (9–12 years old), they already display signs of advanced immune aging, supporting the hypothesis that T cell aging associated with the breakdown of T cell tolerance is not a matter of chronologic age but can occur early in life.

In some autoimmune diseases, CD8 T cells overcome several tolerance mechanisms, exert aberrant effector functions, and cause damage to self-organs. For example, in type 1 diabetes (T1D), beta cell-specific autoreactive CD8 T cells destroy pancreatic beta cells, leading to glucose dysregulation (Bluestone et al., 2010). Beta cell-specific CD8 T cells are also detectable in the peripheral blood of patients with T1D (Wiedeman et al., 2020). Although these autoreactive CD8 T cells express high levels of effector molecules such as granzyme B and IFN-gamma, these CD8 T cells upregulate a combination of inhibitory receptors, including PD-1, LAG3, and TIM3, exhibiting features of exhaustion (Zakharov et al., 2020). Notably, beta cell-specific autoreactive CD8 T cell phenotypes differ by disease progression rate in that beta cell-specific T cells from slow T1D progressors are functionally more exhausted (Wiedeman et al., 2020). Similarly, a previous study showed that a transcriptional signature of CD8 T cell exhaustion is associated with a good prognosis in patients with other autoimmune diseases, including anti-neutrophil cytoplasmic antibody-associated vasculitis and SLE (McKinney et al., 2015). These results support the hypothesis that T cell exhaustion might be a self-protective mechanism to avoid disease progression in autoimmune diseases.

Similar to other immune cells, exhausted T cells are heterogeneous and include progenitor and terminal subpopulations. In chronic viral infection, virus-specific exhausted T cells are composed of at least two subpopulations, memory/stem cell-like progenitor population and terminally differentiated population (Paley et al., 2012). TCF1 (encoded by the gene *TCF7*) has been identified as a critical transcriptional factor for determining the degree of T cell exhaustion (Wu et al., 2016). Virus-specific TCF1^+^ CD8 T cells are a memory/stem cell-like progenitor population, which self-renews and gives rise to terminally differentiated TCF1^low/neg^ T cells (He et al., 2016; Im et al., 2016; Utzschneider et al., 2016; Wu et al., 2016). Notably, these two distinct populations reside in spatially distinct compartments. While TCF1^low/neg^ exhausted T cells tend to reside in non-lymphoid organs, TCF1^+^ progenitor exhausted cells were found in secondary lymphoid organs and restricted predominantly to the T cell zones, the site of low antigen burden and virus replication (Im et al., 2016). TCF1^high^ exhausted T cells, which display self-renewing capacity and are essential for long-term maintenance of persistent T cell responses, have been recently termed as “precursor exhausted T (T_PEX_) cells” and gained much attention (Chen et al., 2019; Utzschneider et al., 2020; Connolly et al., 2021). One of the reasons for this is that this population is the prime target of immunotherapeutic intervention in cancer and infection: T_PEX_ cells, but not terminally differentiated TCF1^low/neg^ exhausted T cells, proliferate in response to PD-1/PD-L1 inhibition (He et al., 2016; Im et al., 2016). Indeed, irrespective of PD-1 expression, the initial frequencies of TCF1^+^ T cells among tumor-infiltrating lymphocytes are closely associated with the duration and efficacy of the response to checkpoint inhibitor blockade in patients with melanoma (Sade-Feldman et al., 2018; Miller et al., 2019). These results suggest the potential of T_PEX_ cells as a predictive marker for a favorable clinical outcome of checkpoint therapy.

Recently, a stem-like exhausted CD8 T cell population in autoimmunity has been identified. Gearty et al., 2021 trace the CD8 T cells that specifically recognize *β* cell protein islet-specific glucose-6-phosphatase catalytic subunit-related protein (IGRP) in the pancreases and pancreatic draining lymph node throughout the course of disease in the non-obese diabetic (NOD) mouse model (Gearty et al., 2021). The investigators have identified a stem-like TCF1^high^ CD8 exhausted T cells in the pancreatic draining lymph node, which can self-renew and give rise to terminally differentiated TCF1^low/neg^ exhausted T cells *in situ*. Then, TCF1^low/neg^ CD8 T cells in the pancreatic draining lymph node migrate to the pancreas, where they undergo further differentiation and destroy β-cells. Importantly, TCF1^high^ CD8 exhausted T cells and the progeny in T1D are functionally and transcriptionally distinct from those in cancers and infections (Gearty et al., 2021). Unlike TCF1^low^ CD8 exhausted T cells in cancers and infections, pancreatic TCF1^low^ CD8 exhausted T cells in T1D do not display a transcriptional or phenotypic exhaustion driven by TOX and retain effector functions despite chronic self-antigen exposure (Gearty et al., 2021). These results may lead to development of new therapeutic approaches for T1D that target a stem-like TCF1^high^ CD8 exhausted T cell pool.

### Aging T Regulatory (Treg) Cells—The Failing Protector

**FIGURE 6:**
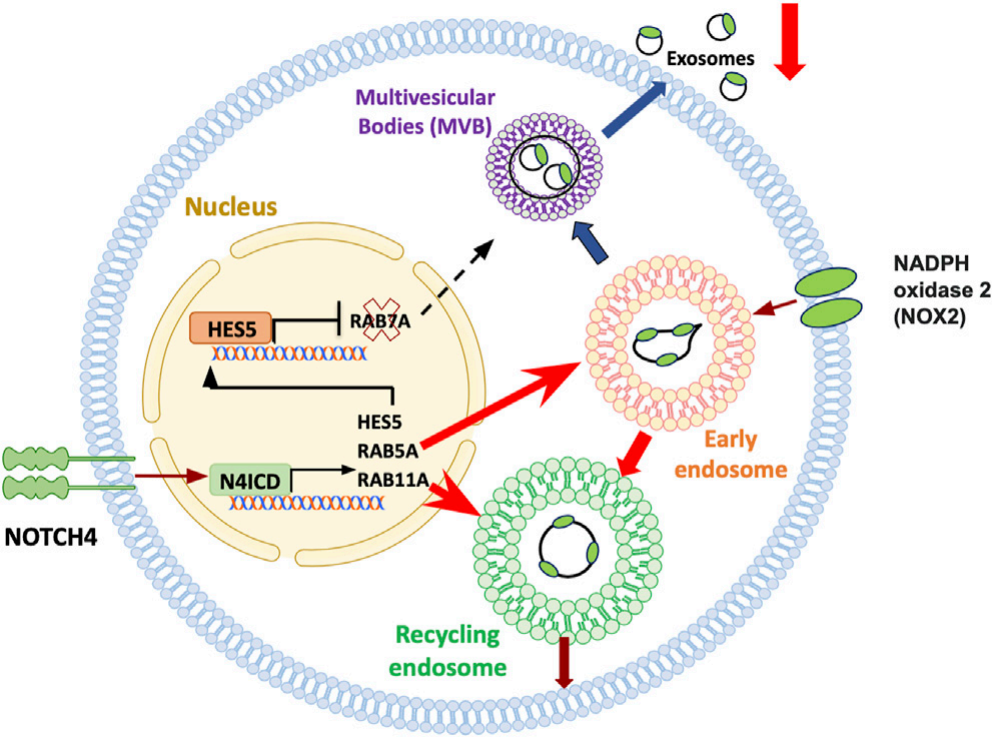
Treg cell failure in aging. Subtle and frank inflammatory states of the aging host have been attributed to a failure of immunosuppressive T regulatory (Treg) cells. Treg cells rely on an array of mechanisms targeting and suppressing effector T cells. CD8^+^ Treg cells function by releasing immunosuppressive exosomes that contain NOX2. Numbers and functional competence of such CD8 Treg cells decline with progressive age in healthy individuals, a process aggravated in the age-associated autoimmune disease giant cell arteritis. In aging CD8^+^ Treg cells, aberrant expression of NOTCH4 redirects the trafficking of intracellular vesicles, essentially disrupting the release of immunosuppressive exosomes. Notch4 signaling targets multiple genes that guard the intracellular vesicle machinery, including RAB5, RAB7 and RAB11. Consequently, early lysosomes are directed towards the recycling pathway, trapping NOX2 in the cell, while formation of multivesicular bodies (MVB) and exosome release are suppressed.

An obvious mechanism through which the aging host becomes susceptible to uncontrolled autoimmunity and tissue inflammation is the failure of immune-inhibitory Treg cells (Figure 6). Both, in secondary lymphoid tissues as well as in peripheral tissue sites, Treg cells function as the guardians of effector T cells and rely on a spectrum of pathways through which they suppress T cell activation, expansion, cytokine production and cytotoxicity (Weyand and Goronzy, 2021). Emerging data support the notion that the pool of activated Treg cells expands with progressing age (Elyahu et al., 2019). However, studies in patients with inflammatory diseases point to a reduction in the suppressive function of Treg cells (Jin et al., 2022). Exposure to the inflammatory environment appears to be relevant for this loss-of-function of Treg cells, as successful treatment often improves Treg cell fitness. How inflammation dismantles immunosuppressive capabilities of Treg cells is unknown but multiple efforts are underway to devise *ex vivo* and *in vivo* approaches enabling the restoration of functional Treg cells in autoimmune disease. In patients with RA, mechanistic explorations have attributed insufficient Treg-mediated dampening of effector T cells to the instability of the lineage-determining transcription factors FOXP3. Specifically, induction of protein phosphatase 1 (PP1) in the inflamed synovium leads to dephosphorylation of Ser418 in the C-terminal DNA-binding domain of FOXP3, disrupting its transcriptional activity and Treg cell stability (Nie et al., 2013). TNF, a key pathogenic cytokine in RA, has been identified as an inducer of PP1, providing a direct connection between maladaptive effector T cell functions and dysfunctional Treg cells.

Age related decline of naive CD8^+^ T-cells has been implicated in the progressive reduction of CD8^+^ Treg cells (Wen et al., 2016). A specialized subset of CD8^+^ Treg cells functions by releasing the enzyme NADPH oxidase 2 (NOX2) packaged in exosomes; such NOX2^+^ CD8^+^ Treg cells steadily decline during the second half of life, as the pool of naive precursor CD8^+^ T-cells shrinks. This process is accelerated in patients with the autoimmune disease GCA. Mechanistic studies have yielded insights into how aging-related signaling pathways weaken CD8^+^ Treg cell function in GCA patients (Figure 6) (Jin K. et al., 2021). Aberrant expression of the cell surface receptor NOTCH4 on patient-derived Treg cells redirects trafficking of intracellular vesicles, therefore controlling the production and release of exosomes. RAB5, RAB11, and RAB7 have emerged as molecules-of-interest, with RAB5 and RAB11 promoting the pool of early and recycling endosomes, essentially trapping NOX2 within the cell. The defect is aggravated by the NOTCH4-dependent repression of RAB7, downregulating the production of multivesicular bodies (MVB) and disrupting the flux of exosomes. Mechanisms underlying the aberrant expression of NOTCH4 on the patient’s CD8^+^ T-cells remain unclear. Notably, aberrant expression of NOTCH receptors is a feature of multiple T-cell subsets in GCA patients, including the expansion of NOTCH1 CD4^+^ T cells, that utilize NOTCH-NOTCH ligand interactions to penetrate the wall of vasa vasora and break the tissue tolerance of large arteries (Wen et al., 2017; Watanabe et al., 2018b).

These data challenge the widely accepted hypothesis that aging is associated with a more robust release of exosomes, which may carry immunosuppressive cargo and thus exacerbate the immunodeficiency of the old adult (Salminen et al., 2020). Damaged tissue may represent an important source of such exosomes, but how the subtle smoldering inflammatory state of the aging host, the remodeling of the immune system and the lack of adequate tissue repair are interweaved will require more granulated investigation.

## Conclusion and Perspectives

Progressive aging of human adults is associated with profound restructuring of the immune system. As a rule, adaptive immunity declines and is replaced by innate immunity, resulting in a bias towards less specific, pro-inflammatory immune responses. During the second half of life, the older adult becomes increasingly immunocompromised, lacking sufficient protection against cancers and infections, often combined with inflammatory disease in a multitude of organ systems. Restructuring of the immune system, including the accumulation of aged T cells, renders the older host susceptible to autoimmunity. Here, we have presented two autoimmune diseases that occur in the setting of immune aging: 1) Rheumatoid arthritis, a T cell-dependent, destructive joint disease. Affected individuals have a signature of premature immune aging best captured by age-inappropriate telomeric loss. 2) Giant Cell Arteritis, a T-cell dependent granulomatous inflammation of large elastic arteries.

In both autoimmune diseases, better understanding of the immune aging process has been instrumental in providing new insights into the pathogenic events driving inflammatory tissue destruction. In RA T cells, most molecular abnormalities are converging at the mitochondrion. Defects in mitochondrial DNA repair lie upstream of several functional outcomes that have direct impact on tissue inflammation. The mitochondrial electron transport chain fails, mitochondrial metabolites are lacking, and low production of reactive oxygen species imposes reductive stress. Deficiency in mitochondrial aspartate production triggers expansion of ER membranes, resulting in hyperproduction of the pro-inflammatory cytokine TNF. TCA cycle reversal promotes the deposition of excess lipids as lipid droplets, which fuel the generation of invasive membrane ruffles. Mis-trafficking of the energy sensor AMPK, caused by deficient posttranslational modification of the kinase, leads to unopposed activation of mTOR on the lysosomal surface. Overall, RA T cells are energy-deprived, tissue-invasive T cells that specialize in the swift production of pro-inflammatory cytokines. T cell phenotypes relevant in RA are tissue invasive, polarized, lipogenic, TNF-secreting, IFN-gamma-producing, short-lived, pyroptosis-prone effector cells. All these phenotypes are enriched among aged T cells. A different set of phenotypes function as disease drivers in GCA. Typically, they aberrantly express NOTCH1, which enables the interaction with NOTCH ligand-expressing endothelial cells, breaking the endothelial barrier and giving T cells access to a protective tissue niche. NOTCH signaling has also been identified as the defect underlying the failure of exosome-producing Treg cells, which dismantles the physiologic break that curtails inappropriate T cell activation.

Several frontiers remain, requiring better insights into molecular events underlying T cell aging and the associated risk of older adults to lose self-tolerance and develop autoimmune tissue inflammation. Specifically, it is unclear how immune aging contributes to different autoimmune conditions, each with a distinct immunopathology. An unresolved question is the tissue patterning of age-related autoimmune diseases. One possibility is that the diverse phenotypes that emerge during the process of T cell aging (Figure 1) have diverse roles as pro-inflammatory drivers. E.g., RA T cells cause inflammation through TNF release and pyroptosis, whereas GCA T cells function by breaking the endothelial barrier. Obviously, T cell aging is a dynamic process, with progressive stages of differentiation, widening the effector profile of aged T cells.

A question that deserves attention is why aged T cells lack responsiveness against pathogens and cancers but are hyperresponders against self-antigens. Part of the opposing behavior of aged T cells may lie in the preferred selection of autoreactive T cell specificities when such T cells are generated by homeostatic T cell proliferation instead of going through the thymus. Homeostatic proliferation, the preferred T cell generation mode in the aging host, depends on the recognition of self-antigens. Accordingly, both in patients with rheumatoid arthritis and in healthy older individuals, the TCR repertoire is contracted and enriched for autoreactive T cells (Wagner et al., 1998; Qi et al., 2014). An alternative hypothesis considers differences in outcome when T cells are stimulated by high-dose viral antigens versus low-affinity autoantigens. The abundance of antigen during viral infection should induce T cell activation and, concomitantly, upregulation of the negative regulatory machinery (e.g., PD-1). Auto-antigen may be sufficiently stimulatory to induce cytokine release but may fail to upregulate sufficient negative regulatory molecules. If primed accordingly, the old T cell would respond to autoantigen with prolonged IFN-gamma and TNF production but would fail to render an effective antiviral response.

Finally, discovering the mechanism underlying the prematurity of immune aging in rheumatoid arthritis could yield valuable information, help define druggable targets and provide tools to quantify immune aging in healthy individuals and patients with autoimmune disease.

## References

[B1] Akbar A. N. Henson S. M. (2011). Are Senescence and Exhaustion Intertwined or Unrelated Processes that Compromise Immunity? Nat. Rev. Immunol. 11 (4), 289–295. 10.1038/nri2959 21436838

[B2] Antonioli L. Pacher P. Vizi E. S. Haskó G. (2013). CD39 and CD73 in Immunity and Inflammation. Trends Mol. Med. 19 (6), 355–367. 10.1016/j.molmed.2013.03.005 23601906 PMC3674206

[B3] Arthur L. Esaulova E. Mogilenko D. A. Tsurinov P. S. Burdess S. Laha A. (2021). Cellular and Plasma Proteomic Determinants of COVID-19 and Non-COVID-19 Pulmonary Diseases Relative to Healthy Aging. Nat. Aging 1 (6), 535–549. 10.1038/s43587-021-00067-x 37117829

[B4] Blank C. U. Haining W. N. Held W. Hogan P. G. Kallies A. Lugli E. (2019). Defining ′T Cell Exhaustion′. Nat. Rev. Immunol. 19 (11), 665–674. 10.1038/s41577-019-0221-9 31570879 PMC7286441

[B5] Bluestone J. A. Herold K. Eisenbarth G. (2010). Genetics, Pathogenesis and Clinical Interventions in Type 1 Diabetes. Nature 464 (7293), 1293–1300. 10.1038/nature08933 20432533 PMC4959889

[B6] Calcinotto A. Kohli J. Zagato E. Pellegrini L. Demaria M. Alimonti A. (2019). Cellular Senescence: Aging, Cancer, and Injury. Physiol. Rev. 99 (2), 1047–1078. 10.1152/physrev.00020.2018 30648461

[B7] Cao W. Fang F. Gould T. Li X. Kim C. Gustafson C. (2020). Ecto-NTPDase CD39 Is a Negative Checkpoint that Inhibits Follicular Helper Cell Generation. J. Clin. Invest. 130 (7), 3422–3436. 10.1172/JCI132417 32452837 PMC7324201

[B8] Chen Z. Ji Z. Ngiow S. F. Manne S. Cai Z. Huang A. C. (2019). TCF-1-Centered Transcriptional Network Drives an Effector versus Exhausted CD8 T Cell-Fate Decision. Immunity 51 (5), 840–855. 10.1016/j.immuni.2019.09.013 31606264 PMC6943829

[B9] Colmegna I. Diaz-Borjon A. Fujii H. Schaefer L. Goronzy J. J. Weyand C. M. (2008). Defective Proliferative Capacity and Accelerated Telomeric Loss of Hematopoietic Progenitor Cells in Rheumatoid Arthritis. Arthritis Rheum. 58 (4), 990–1000. 10.1002/art.23287 18383391 PMC2820283

[B10] Connolly K. A. Kuchroo M. Venkat A. Khatun A. Wang J. William I. (2021). A Reservoir of Stem-like CD8 + T Cells in the Tumor-Draining Lymph Node Preserves the Ongoing Antitumor Immune Response. Sci. Immunol. 6 (64), eabg7836. 10.1126/sciimmunol.abg7836 34597124 PMC8593910

[B11] Del Giudice G. Goronzy J. J. Grubeck-Loebenstein B. Lambert P.-H. Mrkvan T. Stoddard J. J. (2018). Fighting against a Protean Enemy: Immunosenescence, Vaccines, and Healthy Aging. NPJ Aging Mech. Dis. 4, 1. 10.1038/s41514-017-0020-0 29285399 PMC5740164

[B12] Depuydt M. A. C. Prange K. H. M. Slenders L. Örd T. Elbersen D. Boltjes A. (2020). Microanatomy of the Human Atherosclerotic Plaque by Single-Cell Transcriptomics. Circ. Res. 127 (11), 1437–1455. 10.1161/CIRCRESAHA.120.316770 32981416 PMC7641189

[B13] De Bock K. Georgiadou M. Schoors S. Kuchnio A. Wong B. W. Cantelmo A. R. (2013). Role of PFKFB3-Driven Glycolysis in Vessel Sprouting. Cell 154 (3), 651–663. 10.1016/j.cell.2013.06.037 23911327

[B14] Dvergsten J. A. Mueller R. G. Griffin P. Abedin S. Pishko A. Michel J. J. (2013). Premature Cell Senescence and T Cell Receptor-independent Activation of CD8+ T Cells in Juvenile Idiopathic Arthritis. Arthritis Rheum. 65 (8), 2201–2210. 10.1002/art.38015 23686519 PMC3729743

[B15] Elyahu Y. Hekselman I. Eizenberg-Magar I. Berner O. Strominger I. Schiller M. (2019). Aging Promotes Reorganization of the CD4 T Cell Landscape toward Extreme Regulatory and Effector Phenotypes. Sci. Adv. 5 (8), eaaw8330. 10.1126/sciadv.aaw8330 31457092 PMC6703865

[B16] Fang F. Yu M. Cavanagh M. M. Hutter Saunders J. Qi Q. Ye Z. (2016). Expression of CD39 on Activated T Cells Impairs Their Survival in Older Individuals. Cel Rep. 14 (5), 1218–1231. 10.1016/j.celrep.2016.01.002 PMC4851554 26832412

[B17] Franceschi C. Garagnani P. Parini P. Giuliani C. Santoro A. (2018a). Inflammaging: a New Immune-Metabolic Viewpoint for Age-Related Diseases. Nat. Rev. Endocrinol. 14 (10), 576–590. 10.1038/s41574-018-0059-4 30046148

[B18] Franceschi C. Zaikin A. Gordleeva S. Ivanchenko M. Bonifazi F. Storci G. (2018b). Inflammaging 2018: An Update and a Model. Semin. Immunol. 40, 1–5. 10.1016/j.smim.2018.10.008 30392751

[B19] Furman D. Campisi J. Verdin E. Carrera-Bastos P. Targ S. Franceschi C. (2019). Chronic Inflammation in the Etiology of Disease across the Life Span. Nat. Med. 25 (12), 1822–1832. 10.1038/s41591-019-0675-0 31806905 PMC7147972

[B20] Gearty S. V. Dündar F. Zumbo P. Espinosa-Carrasco G. Shakiba M. Sanchez-Rivera F. J. (2021). An Autoimmune Stem-like CD8 T Cell Population Drives Type 1 Diabetes. Nature 602, 156–161. 10.1038/s41586-021-04248-x 34847567 PMC9315050

[B21] Gloor A. D. Berry G. J. Goronzy J. J. Weyand C. M. (2022). Age as a Risk Factor in Vasculitis. Semin. Immunopathology. 10.1007/s00281-022-00911-1 PMC9064861 35141865

[B22] Goronzy J. J. Qi Q. Olshen R. A. Weyand C. M. (2015). High-throughput Sequencing Insights into T-Cell Receptor Repertoire Diversity in Aging. Genome Med. 7 (1), 117. 10.1186/s13073-015-0242-3 26582264 PMC4652363

[B23] Goronzy J. J. Weyand C. M. (2019). Mechanisms Underlying T Cell Ageing. Nat. Rev. Immunol. 19 (9), 573–583. 10.1038/s41577-019-0180-1 31186548 PMC7584388

[B24] Goronzy J. J. Weyand C. M. (2017). Successful and Maladaptive T Cell Aging. Immunity 46 (3), 364–378. 10.1016/j.immuni.2017.03.010 28329703 PMC5433436

[B25] Gustafson C. E. Kim C. Weyand C. M. Goronzy J. J. (2020). Influence of Immune Aging on Vaccine Responses. J. Allergy Clin. Immunol. 145 (5), 1309–1321. 10.1016/j.jaci.2020.03.017 32386655 PMC7198995

[B26] Hashimoto K. Kouno T. Ikawa T. Hayatsu N. Miyajima Y. Yabukami H. (2019). Single-cell Transcriptomics Reveals Expansion of Cytotoxic CD4 T Cells in Supercentenarians. Proc. Natl. Acad. Sci. U.S.A. 116 (48), 24242–24251. 10.1073/pnas.1907883116 31719197 PMC6883788

[B27] He R. Hou S. Liu C. Zhang A. Bai Q. Han M. (2016). Follicular CXCR5-Expressing CD8+ T Cells Curtail Chronic Viral Infection. Nature 537 (7620), 412–416. 10.1038/nature19317 27501245

[B28] Hernandez-Segura A. Nehme J. Demaria M. (2018). Hallmarks of Cellular Senescence. Trends Cel Biol. 28 (6), 436–453. 10.1016/j.tcb.2018.02.001 29477613

[B29] Im S. J. Hashimoto M. Gerner M. Y. Lee J. Kissick H. T. Burger M. C. (2016). Defining CD8+ T Cells that Provide the Proliferative Burst after PD-1 Therapy. Nature 537 (7620), 417–421. 10.1038/nature19330 27501248 PMC5297183

[B30] Jin J. Kim C. Xia Q. Gould T. M. Cao W. Zhang H. (2021a). Activation of mTORC1 at Late Endosomes Misdirects T Cell Fate Decision in Older Individuals. Sci. Immunol. 6 (60), eabg0791. 10.1126/sciimmunol.abg0791 34145066 PMC8422387

[B31] Jin K. Parreau S. Warrington K. Koster M. Berry G. Goronzy J. J. (2022). Regulatory T Cells in Autoimmune Vasculitis. Front. Immunol. 13, 844300. 10.3389/fimmu.2022.844300 35296082 PMC8918523

[B32] Jin K. Wen Z. Wu B. Zhang H. Qiu J. Wang Y. (2021b). NOTCH-induced Rerouting of Endosomal Trafficking Disables Regulatory T Cells in Vasculitis. J. Clin. Invest. 131 (1), e136042. 10.1172/JCI136042 PMC7773364 32960812

[B33] Juno J. A. van Bockel D. Kent S. J. Kelleher A. D. Zaunders J. J. Munier C. M. L. (2017). Cytotoxic CD4 T Cells-Friend or Foe during Viral Infection? Front. Immunol. 8, 19. 10.3389/fimmu.2017.00019 28167943 PMC5253382

[B34] Kim C. Hu B. Jadhav R. R. Jin J. Zhang H. Cavanagh M. M. (2018). Activation of miR-21-Regulated Pathways in Immune Aging Selects against Signatures Characteristic of Memory T Cells. Cel Rep. 25 (8), 2148–2162. 10.1016/j.celrep.2018.10.074 PMC6371971 30463012

[B35] Kim C. Jin J. Ye Z. Jadhav R. R. Gustafson C. E. Hu B. (2021). Histone Deficiency and Accelerated Replication Stress in T Cell Aging. J. Clin. Invest. 131 (11), e143632. 10.1172/JCI143632 PMC8159689 34060486

[B36] Koetz K. Bryl E. Spickschen K. O'Fallon W. M. Goronzy J. J. Weyand C. M. (2000). T Cell Homeostasis in Patients with Rheumatoid Arthritis. Proc. Natl. Acad. Sci. U.S.A. 97 (16), 9203–9208. 10.1073/pnas.97.16.9203 10922071 PMC16846

[B37] Kumar B. V. Connors T. J. Farber D. L. (2018). Human T Cell Development, Localization, and Function throughout Life. Immunity 48 (2), 202–213. 10.1016/j.immuni.2018.01.007 29466753 PMC5826622

[B38] Kuwabara S. Yamaki M. Yu H. Itoh M. (2018). Notch Signaling Regulates the Expression of Glycolysis-Related Genes in a Context-dependent Manner during Embryonic Development. Biochem. Biophysical Res. Commun. 503 (2), 803–808. 10.1016/j.bbrc.2018.06.079 29913146

[B39] Li Y. Goronzy J. J. Weyand C. M. (2018). DNA Damage, Metabolism and Aging in Pro-inflammatory T Cells. Exp. Gerontol. 105, 118–127. 10.1016/j.exger.2017.10.027 29101015 PMC5871568

[B40] Li Y. Shen Y. Hohensinner P. Ju J. Wen Z. Goodman S. B. (2016). Deficient Activity of the Nuclease MRE11A Induces T Cell Aging and Promotes Arthritogenic Effector Functions in Patients with Rheumatoid Arthritis. Immunity 45 (4), 903–916. 10.1016/j.immuni.2016.09.013 27742546 PMC5123765

[B41] Li Y. Shen Y. Jin K. Wen Z. Cao W. Wu B. (2019). The DNA Repair Nuclease MRE11A Functions as a Mitochondrial Protector and Prevents T Cell Pyroptosis and Tissue Inflammation. Cel Metab. 30 (3), 477–492. 10.1016/j.cmet.2019.06.016 PMC7093039 31327667

[B42] Liuzzo G. Goronzy J. J. Yang H. Kopecky S. L. Holmes D. R. Frye R. L. (2000). Monoclonal T-Cell Proliferation and Plaque Instability in Acute Coronary Syndromes. Circulation 101 (25), 2883–2888. 10.1161/01.cir.101.25.2883 10869258

[B43] Liuzzo G. Kopecky S. L. Frye R. L. Fallon W. M. O. Maseri A. Goronzy J. J. (1999). Perturbation of the T-Cell Repertoire in Patients with Unstable Angina. Circulation 100 (21), 2135–2139. 10.1161/01.cir.100.21.2135 10571971

[B44] Liuzzo G. Vallejo A. N. Kopecky S. L. Frye R. L. Holmes D. R. Goronzy J. J. (2001). Molecular Fingerprint of Interferon-γ Signaling in Unstable Angina. Circulation 103 (11), 1509–1514. 10.1161/01.cir.103.11.1509 11257077

[B45] Maehara T. Kaneko N. Perugino C. A. Mattoo H. Kers J. Allard-Chamard H. (2020). Cytotoxic CD4+ T Lymphocytes May Induce Endothelial Cell Apoptosis in Systemic Sclerosis. J. Clin. Invest. 130 (5), 2451–2464. 10.1172/JCI131700 31990684 PMC7190971

[B46] Mattoo H. Mahajan V. S. Maehara T. Deshpande V. Della-Torre E. Wallace Z. S. (2016). Clonal Expansion of CD4+ Cytotoxic T Lymphocytes in Patients with IgG4-Related Disease. J. Allergy Clin. Immunol. 138 (3), 825–838. 10.1016/j.jaci.2015.12.1330 26971690 PMC5014627

[B47] McKinney E. F. Lee J. C. Jayne D. R. W. Lyons P. A. Smith K. G. C. (2015). T-cell Exhaustion, Co-stimulation and Clinical Outcome in Autoimmunity and Infection. Nature 523 (7562), 612–616. 10.1038/nature14468 26123020 PMC4623162

[B48] Miller B. C. Sen D. R. Al Abosy R. Bi K. Virkud Y. V. LaFleur M. W. (2019). Subsets of Exhausted CD8+ T Cells Differentially Mediate Tumor Control and Respond to Checkpoint Blockade. Nat. Immunol. 20 (3), 326–336. 10.1038/s41590-019-0312-6 30778252 PMC6673650

[B49] Mittelbrunn M. Kroemer G. (2021). Hallmarks of T Cell Aging. Nat. Immunol. 22 (6), 687–698. 10.1038/s41590-021-00927-z 33986548

[B50] Mogilenko D. A. Shchukina I. Artyomov M. N. (2021a). Immune Ageing at Single-Cell Resolution. Nat. Rev. Immunol. 1, 15. 10.1038/s41577-021-00646-4 PMC8609266 34815556

[B51] Mogilenko D. A. Shpynov O. Andhey P. S. Arthur L. Swain A. Esaulova E. (2021b). Comprehensive Profiling of an Aging Immune System Reveals Clonal GZMK+ CD8+ T Cells as Conserved Hallmark of Inflammaging. Immunity 54 (1), 99–115. 10.1016/j.immuni.2020.11.005 33271118

[B52] Moskowitz D. M. Zhang D. W. Hu B. Le Saux S. Yanes R. E. Ye Z. (2017). Epigenomics of Human CD8 T Cell Differentiation and Aging. Sci. Immunol. 2 (8), eaag0192. 10.1126/sciimmunol.aag0192 28439570 PMC5399889

[B53] Nakajima T. Goek O. Zhang X. Kopecky S. L. Frye R. L. Goronzy J. J. (2003). De Novo expression of Killer Immunoglobulin-like Receptors and Signaling Proteins Regulates the Cytotoxic Function of CD4 T Cells in Acute Coronary Syndromes. Circ. Res. 93 (2), 106–113. 10.1161/01.RES.0000082333.58263.58 12816883

[B54] Nakajima T. Schulte S. Warrington K. J. Kopecky S. L. Frye R. L. Goronzy J. J. (2002). T-cell-mediated Lysis of Endothelial Cells in Acute Coronary Syndromes. Circulation 105 (5), 570–575. 10.1161/hc0502.103348 11827921

[B55] Nie H. Zheng Y. Li R. Guo T. B. He D. Fang L. (2013). Phosphorylation of FOXP3 Controls Regulatory T Cell Function and Is Inhibited by TNF-α in Rheumatoid Arthritis. Nat. Med. 19 (3), 322–328. 10.1038/nm.3085 23396208

[B56] Niessner A. Sato K. Chaikof E. L. Colmegna I. Goronzy J. J. Weyand C. M. (2006). Pathogen-Sensing Plasmacytoid Dendritic Cells Stimulate Cytotoxic T-Cell Function in the Atherosclerotic Plaque through Interferon-α. Circulation 114 (23), 2482–2489. 10.1161/CIRCULATIONAHA.106.642801 17116765

[B57] Paley M. A. Kroy D. C. Odorizzi P. M. Johnnidis J. B. Dolfi D. V. Barnett B. E. (2012). Progenitor and Terminal Subsets of CD8 + T Cells Cooperate to Contain Chronic Viral Infection. Science 338 (6111), 1220–1225. 10.1126/science.1229620 23197535 PMC3653769

[B58] Peng Y. Mentzer A. J. Liu G. Yao X. Yin Z. Dong D. (2020). Broad and strong Memory CD4+ and CD8+ T Cells Induced by SARS-CoV-2 in UK Convalescent Individuals Following COVID-19. Nat. Immunol. 21 (11), 1336–1345. 10.1038/s41590-020-0782-6 32887977 PMC7611020

[B59] Piggott K. Deng J. Warrington K. Younge B. Kubo J. T. Desai M. (2011). Blocking the NOTCH Pathway Inhibits Vascular Inflammation in Large-Vessel Vasculitis. Circulation 123 (3), 309–318. 10.1161/CIRCULATIONAHA.110.936203 21220737 PMC3056570

[B60] Poon M. M. L. Byington E. Meng W. Kubota M. Matsumoto R. Grifoni A. (2021). Heterogeneity of Human Anti-viral Immunity Shaped by Virus, Tissue, Age, and Sex. Cel Rep. 37 (9), 110071. 10.1016/j.celrep.2021.110071 PMC8719595 34852222

[B61] Pryshchep S. Sato K. Goronzy J. J. Weyand C. M. (2006). T Cell Recognition and Killing of Vascular Smooth Muscle Cells in Acute Coronary Syndrome. Circ. Res. 98 (9), 1168–1176. 10.1161/01.RES.0000220649.10013.5c 16601227

[B62] Qi Q. Liu Y. Cheng Y. Glanville J. Zhang D. Lee J.-Y. (2014). Diversity and Clonal Selection in the Human T-Cell Repertoire. Proc. Natl. Acad. Sci. U.S.A. 111 (36), 13139–13144. 10.1073/pnas.1409155111 25157137 PMC4246948

[B63] Qiu J. Wu B. Goodman S. B. Berry G. J. Goronzy J. J. Weyand C. M. (2021). Metabolic Control of Autoimmunity and Tissue Inflammation in Rheumatoid Arthritis. Front. Immunol. 12, 652771. 10.3389/fimmu.2021.652771 33868292 PMC8050350

[B64] Sade-Feldman M. Yizhak K. Bjorgaard S. L. Ray J. P. de Boer C. G. Jenkins R. W. (2018). Defining T Cell States Associated with Response to Checkpoint Immunotherapy in Melanoma. Cell 175 (4), 998–1013. 10.1016/j.cell.2018.10.038 30388456 PMC6641984

[B65] Salminen A. Kaarniranta K. Kauppinen A. (2020). Exosomal Vesicles Enhance Immunosuppression in Chronic Inflammation: Impact in Cellular Senescence and the Aging Process. Cell Signal. 75, 109771. 10.1016/j.cellsig.2020.109771 32896608

[B66] Sato K. Niessner A. Kopecky S. L. Frye R. L. Goronzy J. J. Weyand C. M. (2006). TRAIL-expressing T Cells Induce Apoptosis of Vascular Smooth Muscle Cells in the Atherosclerotic Plaque. J. Exp. Med. 203 (1), 239–250. 10.1084/jem.20051062 16418392 PMC2118078

[B67] Sato Y. Boor P. Fukuma S. Klinkhammer B. M. Haga H. Ogawa O. (2020). Developmental Stages of Tertiary Lymphoid Tissue Reflect Local Injury and Inflammation in Mouse and Human Kidneys. Kidney Int. 98 (2), 448–463. 10.1016/j.kint.2020.02.023 32473779

[B68] Sato Y. Mii A. Hamazaki Y. Fujita H. Nakata H. Masuda K. (2016). Heterogeneous Fibroblasts Underlie Age-dependent Tertiary Lymphoid Tissues in the Kidney. JCI Insight 1 (11), e87680. 10.1172/jci.insight.87680 27699223 PMC5033938

[B69] Sato Y. Oguchi A. Fukushima Y. Masuda K. Toriu N. Taniguchi K. (2022). CD153/CD30 Signaling Promotes Age-dependent Tertiary Lymphoid Tissue Expansion and Kidney Injury. J. Clin. Invest. 132 (2), e146071. 10.1172/JCI146071 34813503 PMC8759786

[B70] Schmidt D. Goronzy J. J. Weyand C. M. (1996). CD4+ CD7- CD28- T Cells Are Expanded in Rheumatoid Arthritis and Are Characterized by Autoreactivity. J. Clin. Invest. 97 (9), 2027–2037. 10.1172/JCI118638 8621791 PMC507276

[B71] Schönland S. O. Lopez C. Widmann T. Zimmer J. Bryl E. Goronzy J. J. (2003). Premature Telomeric Loss in Rheumatoid Arthritis Is Genetically Determined and Involves Both Myeloid and Lymphoid Cell Lineages. Proc. Natl. Acad. Sci. U.S.A. 100 (23), 13471–13476. 10.1073/pnas.2233561100 14578453 PMC263838

[B72] Scott A. C. Dündar F. Zumbo P. Chandran S. S. Klebanoff C. A. Shakiba M. (2019). TOX Is a Critical Regulator of Tumour-specific T Cell Differentiation. Nature 571 (7764), 270–274. 10.1038/s41586-019-1324-y 31207604 PMC7698992

[B73] Shao L. Fujii H. Colmegna I. Oishi H. Goronzy J. J. Weyand C. M. (2009). Deficiency of the DNA Repair Enzyme ATM in Rheumatoid Arthritis. J. Exp. Med. 206 (6), 1435–1449. 10.1084/jem.20082251 19451263 PMC2715066

[B74] Shao L. Goronzy J. J. Weyand C. M. (2010). DNA‐dependent Protein Kinase Catalytic Subunit Mediates T‐cell Loss in Rheumatoid Arthritis. EMBO Mol. Med. 2 (10), 415–427. 10.1002/emmm.201000096 20878914 PMC3017722

[B75] Shen Y. Wen Z. Li Y. Matteson E. L. Hong J. Goronzy J. J. (2017). Metabolic Control of the Scaffold Protein TKS5 in Tissue-Invasive, Proinflammatory T Cells. Nat. Immunol. 18 (9), 1025–1034. 10.1038/ni.3808 28737753 PMC5568495

[B76] Sikora E. (2015). Activation-induced and Damage-Induced Cell Death in Aging Human T Cells. Mech. Ageing Dev. 151, 85–92. 10.1016/j.mad.2015.03.011 25843236

[B77] Sudre C. H. Murray B. Varsavsky T. Graham M. S. Penfold R. S. Bowyer R. C. (2021). Attributes and Predictors of Long COVID. Nat. Med. 27 (4), 626–631. 10.1038/s41591-021-01292-y 33692530 PMC7611399

[B78] Takemura S. Braun A. Crowson C. Kurtin P. J. Cofield R. H. O’Fallon W. M. (2001a). Lymphoid Neogenesis in Rheumatoid Synovitis. J. Immunol. 167 (2), 1072–1080. 10.4049/jimmunol.167.2.1072 11441118

[B79] Takemura S. Klimiuk P. A. Braun A. Goronzy J. J. Weyand C. M. (2001b). T Cell Activation in Rheumatoid Synovium Is B Cell Dependent. J. Immunol. 167 (8), 4710–4718. 10.4049/jimmunol.167.8.4710 11591802

[B80] Timperi E. Barnaba V. (2021). CD39 Regulation and Functions in T Cells. Ijms 22 (15), 8068. 10.3390/ijms22158068 34360833 PMC8348030

[B81] Utzschneider D. T. Charmoy M. Chennupati V. Pousse L. Ferreira D. P. Calderon-Copete S. (2016). T Cell Factor 1-Expressing Memory-like CD8+ T Cells Sustain the Immune Response to Chronic Viral Infections. Immunity 45 (2), 415–427. 10.1016/j.immuni.2016.07.021 27533016

[B82] Utzschneider D. T. Gabriel S. S. Chisanga D. Gloury R. Gubser P. M. Vasanthakumar A. (2020). Early Precursor T Cells Establish and Propagate T Cell Exhaustion in Chronic Infection. Nat. Immunol. 21 (10), 1256–1266. 10.1038/s41590-020-0760-z 32839610

[B83] Wagner U. G. Koetz K. Weyand C. M. Goronzy J. J. (1998). Perturbation of the T Cell Repertoire in Rheumatoid Arthritis. Proc. Natl. Acad. Sci. U.S.A. 95 (24), 14447–14452. 10.1073/pnas.95.24.14447 9826720 PMC24393

[B84] Watanabe R. Berry G. J. Liang D. H. Goronzy J. J. Weyand C. M. (2020). Cellular Signaling Pathways in Medium and Large Vessel Vasculitis. Front. Immunol. 11, 587089. 10.3389/fimmu.2020.587089 33072134 PMC7544845

[B85] Watanabe R. Hilhorst M. Zhang H. Zeisbrich M. Berry G. J. Wallis B. B. (2018a). Glucose Metabolism Controls Disease-specific Signatures of Macrophage Effector Functions. JCI Insight 3 (20), e123047. 10.1172/jci.insight.123047 PMC6237479 30333306

[B86] Watanabe R. Maeda T. Zhang H. Berry G. J. Zeisbrich M. Brockett R. (2018b). MMP (Matrix Metalloprotease)-9-Producing Monocytes Enable T Cells to Invade the Vessel Wall and Cause Vasculitis. Circ. Res. 123 (6), 700–715. 10.1161/CIRCRESAHA.118.313206 29970365 PMC6202245

[B87] Watanabe R. Shirai T. Namkoong H. Zhang H. Berry G. J. Wallis B. B. (2017). Pyruvate Controls the Checkpoint Inhibitor PD-L1 and Suppresses T Cell Immunity. J. Clin. Invest. 127 (7), 2725–2738. 10.1172/JCI92167 28604383 PMC5490755

[B88] Wen Z. Jin K. Shen Y. Yang Z. Li Y. Wu B. (2019). N-myristoyltransferase Deficiency Impairs Activation of Kinase AMPK and Promotes Synovial Tissue Inflammation. Nat. Immunol. 20 (3), 313–325. 10.1038/s41590-018-0296-7 30718913 PMC6396296

[B89] Wen Z. Shen Y. Berry G. Shahram F. Li Y. Watanabe R. (2017). The Microvascular Niche Instructs T Cells in Large Vessel Vasculitis *via* the VEGF-Jagged1-Notch Pathway. Sci. Transl. Med. 9 (399), eaal3322. 10.1126/scitranslmed.aal3322 28724574 PMC5708299

[B90] Wen Z. Shimojima Y. Shirai T. Li Y. Ju J. Yang Z. (2016). NADPH Oxidase Deficiency Underlies Dysfunction of Aged CD8+ Tregs. J. Clin. Invest. 126 (5), 1953–1967. 10.1172/JCI84181 27088800 PMC4855948

[B91] Weyand C. M. Brandes J. C. Schmidt D. Fulbright J. W. Goronzy J. J. (1998). Functional Properties of CD4+ CD28- T Cells in the Aging Immune System. Mech. Ageing Dev. 102 (2-3), 131–147. 10.1016/s0047-6374(97)00161-9 9720647

[B92] Weyand C. M. Berry G. J. Goronzy J. J. (2018a). The Immunoinhibitory PD-1/pd-L1 Pathway in Inflammatory Blood Vessel Disease. J. Leukoc. Biol. 103 (3), 3MA0717–283. 10.1189/jlb.3MA0717-283 PMC6457250 28848042

[B93] Weyand C. M. Goronzy J. J. (2003). Giant-cell Arteritis and Polymyalgia Rheumatica. Ann. Intern. Med. 139 (6), 505–515. 10.7326/0003-4819-139-6-200309160-00015 13679329

[B94] Weyand C. M. Goronzy J. J. (2014). Giant-Cell Arteritis and Polymyalgia Rheumatica. N. Engl. J. Med. 371 (1), 50–57. 10.1056/NEJMcp1214825 24988557 PMC4277693

[B95] Weyand C. M. Goronzy J. J. (2013). Immune Mechanisms in Medium and Large-Vessel Vasculitis. Nat. Rev. Rheumatol. 9 (12), 731–740. 10.1038/nrrheum.2013.161 24189842 PMC4277683

[B96] Weyand C. M. Goronzy J. J. (2017). Immunometabolism in Early and Late Stages of Rheumatoid Arthritis. Nat. Rev. Rheumatol. 13 (5), 291–301. 10.1038/nrrheum.2017.49 28360422 PMC6820517

[B97] Weyand C. M. Goronzy J. J. (2020). Immunometabolism in the Development of Rheumatoid Arthritis. Immunol. Rev. 294 (1), 177–187. 10.1111/imr.12838 31984519 PMC7047523

[B98] Weyand C. M. Goronzy J. J. (2021). The Immunology of Rheumatoid Arthritis. Nat. Immunol. 22 (1), 10–18. 10.1038/s41590-020-00816-x 33257900 PMC8557973

[B99] Weyand C. M. Shen Y. Goronzy J. J. (2018b). Redox-sensitive Signaling in Inflammatory T Cells and in Autoimmune Disease. Free Radic. Biol. Med. 125, 36–43. 10.1016/j.freeradbiomed.2018.03.004 29524605 PMC6128787

[B100] Weyand C. M. Zeisbrich M. Goronzy J. J. (2017). Metabolic Signatures of T-Cells and Macrophages in Rheumatoid Arthritis. Curr. Opin. Immunol. 46, 112–120. 10.1016/j.coi.2017.04.010 28538163 PMC5554742

[B101] Wherry E. J. Kurachi M. (2015). Molecular and Cellular Insights into T Cell Exhaustion. Nat. Rev. Immunol. 15 (8), 486–499. 10.1038/nri3862 26205583 PMC4889009

[B102] Whiteside S. K. Snook J. P. Williams M. A. Weis J. J. (2018). Bystander T Cells: A Balancing Act of Friends and Foes. Trends Immunol. 39 (12), 1021–1035. 10.1016/j.it.2018.10.003 30413351 PMC6269193

[B103] Wiedeman A. E. Muir V. S. Rosasco M. G. DeBerg H. A. Presnell S. Haas B. (2019). Autoreactive CD8+ T Cell Exhaustion Distinguishes Subjects with Slow Type 1 Diabetes Progression. J. Clin. Invest. 130 (1), 480–490. 10.1172/JCI126595 PMC6934185 31815738

[B104] Williamson E. J. Walker A. J. Bhaskaran K. Bacon S. Bates C. Morton C. E. (2020). Factors Associated with COVID-19-Related Death Using OpenSAFELY. Nature 584 (7821), 430–436. 10.1038/s41586-020-2521-4 32640463 PMC7611074

[B105] Wu B. Qiu J. Zhao T. V. Wang Y. Maeda T. Goronzy I. N. (2020). Succinyl-CoA Ligase Deficiency in Pro-inflammatory and Tissue-Invasive T Cells. Cel Metab. 32 (6), 967–980. 10.1016/j.cmet.2020.10.025 PMC7755381 33264602

[B106] Wu B. Zhao T. V. Jin K. Hu Z. Abdel M. P. Warrington K. J. (2021). Mitochondrial Aspartate Regulates TNF Biogenesis and Autoimmune Tissue Inflammation. Nat. Immunol. 22 (12), 1551–1562. 10.1038/s41590-021-01065-2 34811544 PMC8756813

[B107] Wu T. Ji Y. Moseman E. A. Xu H. C. Manglani M. Kirby M. (2016). The TCF1-Bcl6 axis Counteracts Type I Interferon to Repress Exhaustion and Maintain T Cell Stemness. Sci. Immunol. 1 (6), eaai8593. 10.1126/sciimmunol.aai8593 28018990 PMC5179228

[B108] Xie Y. Xu E. Bowe B. Al-Aly Z. (2022). Long-term Cardiovascular Outcomes of COVID-19. Nat. Med. 28, 583–590. 10.1038/s41591-022-01689-3 35132265 PMC8938267

[B109] Yang Z. Fujii H. Mohan S. V. Goronzy J. J. Weyand C. M. (2013). Phosphofructokinase Deficiency Impairs ATP Generation, Autophagy, and Redox Balance in Rheumatoid Arthritis T Cells. J. Exp. Med. 210 (10), 2119–2134. 10.1084/jem.20130252 24043759 PMC3782046

[B110] Yang Z. Shen Y. Oishi H. Matteson E. L. Tian L. Goronzy J. J. (2016). Restoring Oxidant Signaling Suppresses Proarthritogenic T Cell Effector Functions in Rheumatoid Arthritis. Sci. Transl. Med. 8 (331), 331ra338. 10.1126/scitranslmed.aad7151 PMC5074090 27009267

[B111] Zakharov P. N. Hu H. Wan X. Unanue E. R. (2020). Single-cell RNA Sequencing of Murine Islets Shows High Cellular Complexity at All Stages of Autoimmune Diabetes. J. Exp. Med. 217 (6), e20192362. 10.1084/jem.20192362 32251514 PMC7971127

[B112] Zhang H. Watanabe R. Berry G. J. Nadler S. G. Goronzy J. J. Weyand C. M. (2019). CD28 Signaling Controls Metabolic Fitness of Pathogenic T Cells in Medium and Large Vessel Vasculitis. J. Am. Coll. Cardiol. 73 (14), 1811–1823. 10.1016/j.jacc.2019.01.049 30975299 PMC6709860

[B113] Zhang H. Watanabe R. Berry G. J. Tian L. Goronzy J. J. Weyand C. M. (2018). Inhibition of JAK-STAT Signaling Suppresses Pathogenic Immune Responses in Medium and Large Vessel Vasculitis. Circulation 137 (18), 1934–1948. 10.1161/CIRCULATIONAHA.117.030423 29254929 PMC5930040

[B114] Zhao Y. Shao Q. Peng G. (2020). Exhaustion and Senescence: Two Crucial Dysfunctional States of T Cells in the Tumor Microenvironment. Cell Mol Immunol 17 (1), 27–35. 10.1038/s41423-019-0344-8 31853000 PMC6952436

